# The impact of different benefit packages of Medical Financial Assistance Scheme on health service utilization of poor population in Rural China

**DOI:** 10.1186/1472-6963-10-170

**Published:** 2010-06-17

**Authors:** Yanhua Hao, Qunhong Wu, Zhenzhong Zhang, Lijun Gao, Ning Ning, Mingli Jiao, David Zakus

**Affiliations:** 1 Department of Social Medicine, School of Health Management, Harbin Medical University. 157 Baojian Road, Harbin City, Heilongjiang Province, China; 2 National Health Economic Institute, Ministry of Health, China, 38 Xueyuan Road, Haidian District, Beijing, China; 3 Center for International Health, Dalla Lana School of Public Health, University of Toronto, Canada

## Abstract

**Background:**

Since 2003 and 2005, National Pilot Medical Financial Assistance Scheme (MFA) has been implemented in rural and urban areas of China to improve the poorest families' accessibility to health services. Local governments of the pilot areas formulated various benefit packages. Comparative evaluation research on the effect of different benefit packages is urgently needed to provide evidence for improving policy-making of MFA. This study was based on a MFA pilot project, which was one component of Health VIII Project conducted in rural China. This article aimed to compare difference in health services utilization of poor families between two benefit package project areas: H8 towns (package covering inpatient service, some designated preventive and curative health services but without out-patient service reimbursement in Health VIII Project,) and H8SP towns (package extending coverage of target population, covering out- patient services and reducing co-payment rate in Health VIII Supportive Project), and to find out major influencing factors on their services utilization.

**Methods:**

A cross-sectional survey was conducted in 2004, which used stratified cluster sampling method to select poor families who have been enrolled in MFA scheme in rural areas of ChongQing. All family members of the enrolled households were interviewed. 748 and 1129 respondents from two kinds of project towns participated in the survey. Among them, 625 and 869 respondents were included (age≥15) in the analysis of this study. Two-level linear multilevel model and binomial regressions with a log link were used to assess influencing factors on different response variables measuring service utilization.

**Results:**

In general, there was no statistical significance in physician visits and hospitalizations among all the respondents between the two kinds of benefit package towns. After adjusting for major confounding factors, poor families in H8SP towns had much higher frequency of MFA use (β = 1.17) and less use of hospitalization service (OR = 0.7 (H8SP/H8), 95%CI (0.5, 1.0)) among all the respondents. While calculating use of hospital services among those who needed, there was significant difference (p = 0.032) in percentage of hospitalization use between H8SP towns (46%) and H8 towns (33%). Meanwhile, the non-use but ought-to-use hospitalization ratio of H8SP (54%) was lower than that of H8 (67 %) towns. This indicated that hospitalization utilizations had improved in H8SP towns among those who needed. Awareness of MFA detailed benefit package and presence of physician diagnosed chronic disease had significant association with frequency of MFA use and hospitalizations. There was no significant difference in rate of borrowing money for illness treatment between the two project areas. Large amount of medical debt had strong association with hospitalization utilization.

**Conclusions:**

The new extended benefit package implemented in pilot towns significantly increased the poor families' accessibility to MFA package in H8SP than that of H8 towns, which reduced poor families' demand of hospitalization services for their chronic diseases, and improved the poor population's utilization of out-patient services to some degree. It can encourage poor people to use more outpatient services thus reduce their hospitalization need. Presence of chronic disease and hospitalization had strong association with the presence of large amount of medical debt, which indicated that: although establishment of MFA had facilitated accessibility of poor families to this new system, and improved service utilization of poor families to some degree, but its role in reducing poor families' medical debt resulted from chronic disease and hospitalization was still very limited. Besides, the following requirements of MFA: co-payment for in-patient services, ceiling and deductibles for reimbursement, limitations on eligibility for diseases reimbursement, also served as most important obstacles for poor families' access to health care.

Therefore, there is great need to improve MFA benefit package design in the future, including extending to cover out-patient services, raising ceiling for reimbursement, removing deductibles of MFA, reducing co-payment rate, and integrating MFA with New Rural Cooperative Medical Scheme more closely so as to provide more protection to the poor families.

## Background

Poverty is a worldwide issue that requires arduous efforts of governments to address. The relationship between poverty and health has been well documented [1-4]. To break the vicious cycle between poverty and poor health through investment in human capital and health service has been a rational choice of poverty reduction strategies [5-7]. The Chinese government has also made tremendous efforts to carry out all sorts of poverty alleviation strategies, and now gradually re-establish the universal health insurance system for rural and urban residents [8].

Since 1980s, China had moved towards a market economy, this trend was also reflected in health system [8,9]. In rural areas, the transition from agricultural collectives to what was termed as the 'Household responsibility system' weakened the financial base of the Cooperative Medical Scheme (CMS), which contributing to its collapse in most rural communities. CMS insurance coverage for rural residents had fallen to12.8% in 1993, and 6.6 % in 1998, respectively [10]. Percentage of rural residents without any insurance was 87.3% in 1998 [11]. User charges and high direct cost blocked the access of many rural residents for lack of sufficient income to purchase basic health care when needed. Moreover, medical expenses also caused financial catastrophe for many rural families as most rural residents had to pay for services by out-of pocket [12,13]. Concerned with the increasingly deteriorated health situation in rural areas, Chinese government has decided to establish Medical Financial system (MFA) and New Cooperative Medical Scheme (NCMS) for rural population since late 2002 and 2003 respectively.

Unlike the CMS scheme which has existed long time ago, MFA is a brand new system in China, which was first put forwarded by the Health VIII project titled Strengthening Basic Rural Health Care in China, which was launched in 1998 under the joint support of World Bank and Chinese government. It was comprehensive poverty alleviation project included MFA Scheme, and targeted at poor rural areas. The goals of this scheme were to explore setting up health security system which directly targeting at the poorest, find out an effective way of improving their accessibility and overall health status of poor populations, facilitate poverty reduction and promote sustainable development in rural areas [14].

Although the design of MFA of Health VIII Project (H8) had very clear objectives and very good intention, the early stage of MFA implementation was unsatisfactory mainly due to its very low service utilization. With the support of DFID (Department for International Development of UK), a further pilot activity, Health VIII Supportive Project (H8SP), was designed and implemented in 2000 to reduce factors obstructing accessibility of the poorest to MFA.

In poor rural areas, the exact proportions of poor people lived under absolute poverty line is not clear in fact. A study reported [15] that the proportion of extremely poor people in project counties ranged from 7.89%~16.41% during 1992~1993, and there was 58% of the population in these counties with annual per capita income lower than 500 RMB. One report of World Bank showed that, in the 592 poorest counties identified by Office of Poverty Alleviation and Development of the State Council, at least half of the population lived under the absolute poverty line [16]. An other study reported that, In 2000, an average 18.6% of rural population lived under the national poverty line (640 RMB per capita income) in these project counties and that meant the 5% target population coverage of MFA only covered 37% of the poor population who needed protection of MFA [17,18].

In addition, extreme poverty status of the poorest families seriously limited their ability to offer any co-payment for health services. Many poor families had to owe debt to clinics or borrow money from others to pay for their services. In fact, 75% of Medical cost occurred in the village clinics, but H8 package didn't cover out-patient services [15]. Original benefit package only covered in-patient service, and set up 40-60% co-payment rate. The high co-payment rate was far beyond their affordability.

The following issues: payment method of fee for service to the services providers, setting up ceiling for reimbursement to avoid risk of MFA fund, lack of effective measures to control the provider's behaviour for profit, would contribute to the very low MFA utilization. After one year implementation of MFA in the project areas, only 279 persons in Wuxi County had utilized MFA package among the 31976 MFA cardholders, the percentage of MFA use was only 0.87% and hospitalization rate was lower than 1% among the poorest population [19].

Due to this unsatisfactory health services utilization, a new design of benefit package was conducted by the joint efforts of project staff of these pilot project areas, national experts of MFA, Foreign Loan Office of Ministry of Health and DFID to improve health services utilization of MFA cardholders [19].

This study was based on the MFA pilot project activities funded by DFID, and aimed at comparing difference in services utilization and exploring major influencing factors on health service use of poor MFA enrollers between original benefit package and new package MFA project areas.

Thus, there were two kinds of project areas in this study: one defined as H8 towns acted as control group, which carried out the original benefit package, the other was treatment group, H8SP towns, which implemented new designed benefit package. The original designated coverage of MFA was 5% of rural population of project areas, and benefit package included paying for preventive and curative services, such as prenatal and postnatal care, clean delivery, free vaccine inoculation for children; 40-70% reimbursement of inpatient expenditure, and helping MFA cardholders to join NCMS (rural New Cooperative Medical Scheme) by paying for their premium if the town had implemented NCMS. In addition to the original benefit of H8, the new benefit package of H8SP enlarged coverage of MFA from 5% to 8%~11.3% of rural population in pilot project towns, extended to cover the following services: providing outpatient service reimbursement to the enrollee, increasing reimbursement rate for MFA in-patient expenditure to 60~80%, further strengthening financial support to those vulnerable MFA cardholders.

**Figure 1:**
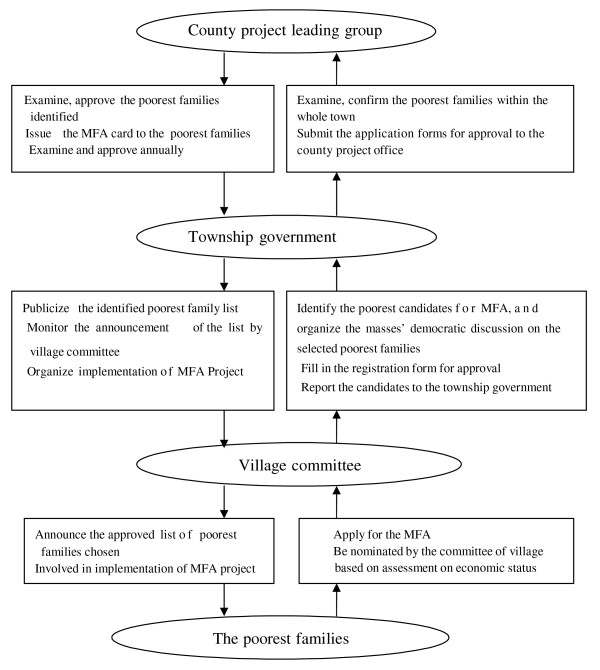
**The Procedure of Identifying the Target Poorest Families**. Text in the oval indicates the different level organizations and population involved in the process of identifying target poor families. Text in the rectangle shows their roles and responsibility during the process.

The MFA target populations were selected based on household income level in terms of strict procedure set by MFA scheme (Figure 1). The poor families eligible for MFA scheme were those lived under the national poverty line [18]. The committee of the village was concretely responsible for identifying target families. First, necessary publicity on MFA policy should be carried out and the potential eligible families could apply for MFA voluntarily. In practice, many candidates were nominated by committee of villagers, based on family's economic assessment to figure out real economic situation of the candidate families, and listed the poorest family names in terms of the order of their poverty degree. Then the committee organized the masses' democratic discussion on the name list of poorest families selected. The candidate families list was reported to township government for examining and confirming, and then reported to county project office for approval. Once approved, the name list should be publicized for the purpose of exposure to democratic supervision of the villagers. In order to make sure the poorest families could be covered by MFA scheme, the enrollees of MFA would accept review once a year [14]. The target families were issued a special MFA card, which acted as their identification for utilizing MFA benefit package. Services they received were recorded on the card. The new benefit package of MFA was implemented in the pilot counties and towns since late 2000.

## Methods

### Study population

The data were collected from a cross-sectional survey conducted in October, 2004. We identified ChongQing municipality as the study site, which was one of the seven provinces implemented health VIII project in China. Stratified cluster sampling method was adopted. Two pilot counties, Wuxi (relatively poor) and Qianjiang (relatively wealthy) were selected in terms of their socio- economic level and implementation of MFA project. Both of them had implemented the project stably. In each county, four towns were selected, among them, two towns with relatively better off economic level were selected but carried out H8 and H8SP benefit package respectively, while in the other relatively poor towns, also one H8 town and one H8SP town were selected. Overall, eight towns were selected in two pilot counties, among them four towns conducted H8 benefit package, the other four towns carried out H8SP package. In each sample town, five villages were identified in terms of socio-economic level and distance to the town centre. All households enrolled by MFA project in each of sample villages, were automatically identified as survey subjects. All family members of enrolled households were interviewed face to face. In- depth interviews were carried out with the local key informants of MFA to collect more detailed and extensive information on implementation of MFA. This study was approved by Medical Ethic Committee of Harbin Medical University. Interviewers were trained before the survey and obtained oral consent from respondents before interviewing.

In total, 671 households, 1877 individuals were interviewed, among them, 324 households, 748 individuals resided in H8 towns and 347 households, 1129 individuals in H8SP towns. This survey response rate was 94%. Excluding the respondents with missing data (6 individuals in H8 towns and 11 in H8SP towns) and respondents aged lower than 15 years old (117 in H8 towns and 249 in H8SP towns), 625 respondents in H8 towns and 869 respondents in H8SP towns were included in analysis of the study eventually.

### Dependent variables

Dependent variables indicated outcome of target population's use of health services. Five variables were included in this study: physician visits within the last two weeks; hospitalizations within the last year; frequency of MFA use within the last year; percent and reasons for non- visiting a physician among people who reported their illness in the last two weeks and percent and reasons for non-use but ought-to-use hospitalization within the last year.

We measured these variables with the following questions: (1) "Have you visited a physician for your illness within the last two weeks before this survey"; (respondents were grouped into two categories, physician users and non-physician users in terms of their reply); (2) "How many times have you been hospitalized during the last year?" (Respondents who reported one or more hospitalizations within the last year were categorized into hospital user);

(3) "How many times in last year did a physician recommend you to be hospitalized, but you refused because of various reasons?" (Respondents who answered positively to this question were categorized into non-hospitalization users who ought to utilize. (4) "How many times have you used your MFA card in last year?" The high-frequency MFA users were defined as users who utilized their MFA card two or more times in the last year. This question measured the total use of services that received reimbursement by MFA. (5) "Why didn't you go to see a doctor for your illness in the last two weeks; and why didn't you go to the hospital when the physician recommended you should utilize hospitalization?" This question aimed to find out reasons why MFA cardholders refused to use services.

In addition, another dependent variable--medical debt rate was introduced in the study to indirectly measure the effect of MFA benefit package on financial burden of MFA cardholder paying for health care. This variable was measured by question "Did you borrow money to pay for your family members' illness treatment in the last year? If yes, how much money did you borrowed?" Respondents were dichotomized into two groups based on the amount they borrowed in the last year.

### Independent variables

Independent variables were selected based on Anderson Behaviour Model of health service unitization [20-22]. This model has been extensively employed to explain health care access and utilization [23-25]. It proposes three categories of factors that influence people to utilize health services: predisposing characteristics; enabling resources and the need. Predisposing characteristics reflect individual aspects and are standard measures of socio-demographic attributes: gender, age, marital status, education, etc.

Enabling factors are the personal resources available to an individual that enable or impede the use of health services [20]. In this study, four variables were specified as enabling factors: type of benefit packages they were insured, which were indirectly measured by H8SP/H8 project towns; distance to nearest designated medical centre; awareness of MFA detailed package, financial capacity to afford services. Awareness of MFA detailed package was assessed by the degree that respondents understand contents of benefit package they were insured, and was dichotomized as "1" (knew all, and most of the contents, "0" (knew part, little and nothing). Income is an ideal variable to measure the financial capacity of family to afford health services and economic status. Because all of the subjects in this study lived under the governmental poverty line (annual per capital income lower than 640 RMB), and there were no significant income difference among them, so we adopted a simple and more sensitive variable "availability of food for subsistence" to measure their income status, and this variable was measured by asking the question: "Did your family have enough subsistence food to meet the survival need in the last year?" Six answers were provided: ①Food absolutely rely on government relief or loan.②Most of food rely on government relief or loan.③Part of food rely on government relief or loan.④ Food just enough for a household to make living. ⑤Food can meet the need of the whole family and have some surplus.⑥Food have surplus and can feed some domestic animals. Respondents with the first three answers were categorized into the "extremely poor" group, which had much low capacity to afford health services, the others were classified into the "relatively poor", and they had relatively better economic status.

Need exists when an individual utilize health care based on their recognition of an illness, and it is an important precondition for health services. We specified the disabled status, presence of illness within 2 weeks before the survey and presence of chronic diseases as the need factors in the study. Presence of chronic disease referred to disease diagnosed by a physician in the last six months before survey, or chronic disease that was diagnosed more than six months before survey but recurred within the last six months and received treatment [26].

### Statistical analysis

Inferential statistics were used to test differences in characteristics of two sample populations. Reasons for non-use of health services were compared. Multilevel model and multiple regression models were used to assess influencing factors on dependent variables.

In terms of the hierarchical sampling process used in this study, multilevel modeling strategy is more appropriate to analyze the data, According to principle of applying of multilevel

Model [27], ICC (Intra-class Correlation Coefficient) should be calculated first to identify if between-group heterogeneity exist among the different township groups. If ICC is significant, multi-level model should be adopted. Otherwise, general multiple regression model should be used.[27,28] The results of calculated ICC respectively showed that: Only ICC of frequency of MFA use was significant in this study, which indicate multilevel model should be adopted while other dependent variables could be fitted with general multiple regression models. Binominal regression with a log link was fitted for these binary response variables of hospitalization rate, physician visit rate and medical debt rate respectively [29].

Tabachnick and Fidell suggested a formula for determining the number of predictors in a regression model. They recommend that sample size (N) should be equal to or greater than 50+8 m, where m was the number of independent variables [30]. This criterion was met in the sample for analysis of physician visit rate (N = 190, m = 10), and more than satisfied with the samples for analysis of hospitalization and medical debt rate (N = 1494, m = 11). In these models, main effect of all independent variables listed in the table were included to compare their effects on services utilization after adjusting for other independent variables.

Necessary data processing was conducted to meet the requirements of multilevel modeling. Normalization for variable of frequency of MFA by Log conversion and Grand-mean centering for variable age and distance were conducted. With individual characteristics at the first level and township variable (Type of MFA benefit package) at the second level, Two-level Linear Multilevel models were fitted in terms of the following modeling strategy [27,28,31].

First, analysis began with the estimation of a 'null' or 'intercept-only' model, which contained no predictor variables. This model was used to partition total variance into it's within- and between group components, and also provided baselines for comparing model fit. Second, Contextual variable (second level) was introduced into the model to explain between-group variation. Third, individual level variables (gender, age, marital status, education, distance to medical centre, awareness of MFA package, economic status, disability status, presence of illness in last 2 weeks and chronic disease) were added into model as fixed effect. Fourth, random slope coefficients of individual level were tested. Fifth, Cross-level interactions were assessed. Fixed effects were used unless the test indicated that a random effect significantly improved model fit (P < 0.05). Lastly, the final model was identified based on model comparison.

SAS version 9.1 was used for all analyses, including PROC MIXED for continuous measure of frequency of MFA use, and PROC GENMOD for binary response variables.

## Results

### Characteristics of Samples

The two sample populations had a high proportion of elders aged over 60 (Table 1). Illiteracy accounted for 54% and 38% of samples in H8 and H8SP towns respectively, and male were more than female inH8 towns. The aggregated proportion of unmarried, widowed and divorced people accounted for 33% and 25% in H8 and H8SP. In addition, the poor people of H8 had a higher rate of disability than that of H8SP, which accounted for 29% and 15%, respectively. Respondents in H8 towns with a distance to nearest designated medical centre ≤ 3 km were significantly less than that of H8SP (36% vs. 61%). Awareness of MFA package in H8 towns was much lower than that of H8SP (13% vs. 47%). Economic status of the two groups had significant difference. Extremely poor persons accounted for 36% of the respondents in H8, whereas 19% in H8SP towns. Prevalence rate of illness within 2 weeks before the survey and prevalence rate of chronic disease showed no statistical difference between the two samples.

**Table 1:** Characteristics of sample population (age ≥15) in two project areas

Variables towns (%)	H8 towns (%)	H8 towns (%)	P
	N = 625	N = 869	
**Predisposing factors**			
Age 15-39.9	177 ( 28 )	329 (38)	P < 0.001
40-59.9	188 (30)	273 (31)	
60+	260 (42)	267 (31)	
Gender, male	363 (58)	440 (50)	P = 0.005
Marital status			P = 0.002
Married	417 (67)	649 (75)	
Unmarried	133 (21)	150 (17)	
Divorced	68 (11)	67 (8)	
Widowed	7 (1)	3 (0.3)	
Education			P < 0.001
Illiterate	339(54)	327 (38)	
Primary school	218(35)	365 (42)	
Junior high school	57(9)	159 (18)	
Senior high school	11(2)	18 (2)	
**Enabling factors**			
Distance from home to nearest designated medical centre, ≤ 3 km	224(36)	534 (61)	P < 0.001
Awareness of MFA detailed package	84(13)	410 (47)	P < 0.001
Economic status, extremely poor	225(36)	164 (19)	P < 0.001
**Need factors**			
Disability status, disabled	179 (29)	130 (15)	P < 0.001
Presence of illness in last 2 weeks	85 (14)	105 (12)	P = 0.385
Presence of physician diagnosed chronic disease	176(28)	244 (28)	P = 0.972
**Outcomes**			
Physician visit rate of respondents in last 2 Weeks	56 (9)	78 (9)	P = 0.992
Hospitalization rate	50 (8)	54 (6)	P = 0.181
High-Frequency use of MFA (≥2)	21 (3)	427 (49)	P < 0.000
Percentage of borrowing money for illness treatment (presence/absence)	148(24)	189(22)	P = 0.380
Percentage of Borrowing large amount money for illness treatment (≥500 RMB)	82(13)	108(12)	P = 0.695

Note: Chi- square test was performed using SAS PROC FREQ.

### Health service utilization

Table 2 presents the health services utilization of the two groups. There was no statistical significance in physician visits in the last two weeks and hospitalization rate among all the respondents between two samples, while there was significance difference in the percentage of use among those respondents who need hospitalization service between H8SP towns (46%) and H8 towns (33%). Whereas, non-use but ought-to-use hospitalization rate of H8SP (54%) was lower than that of H8 (67%). This indicated that hospitalization service improved in H8SP towns to some degree. Besides, table 1 shows that high-frequency rate of MFA use in H8SP towns (49%) was significantly higher than that of H8 (3%); the average frequency of MFA use in H8SP was substantially higher than that of H8.

**Table 2:** Comparison of health service utilization between the two project areas (%)

Services utilization		H8	H8SP	P value	Power
		N = 625 (%)	N = 869 (%)		(1-β)
Out-patient Services	Physician visits among all respondents	56 (9)	78 (9)	P = 0.992	0.975
	Physician visits among the respondents with illness in last 2 weeks	56/85 ( 66)	78/105 (74)	P = 0.207	0.77
	Non-visiting physician rate among the respondents with illness in last 2 weeks	29/85 (34)	27/105 (26)	P = 0.207	0.77

Hospitalization Services	Hospitalization rate among all respondents	50 (8)	54 (6)	P = 0.181	0.68
	Hospitalization rate among those needed	50/(103+50) (33)	54/(64+54) (46)	P = 0.032	0.60
	non-use but ought-to-use hospitalization rate	103/(103+50) (67)	64/(64+54) (54)	P = 0.032	0.60

MFA use	Frequencies of MFA use				
	Mean	0.1±0.5	1.9±2.1	P < 0.000	0.83
	Median	0	1		

Note: Non-use but ought-to-use hospitalization rate = no user/(user + non-user who should use)*100%

Chi- square test was performed using SAS PROC FREQ and t-test was performed using SAS PROC TTEST

### Reasons for no-use of health services

Among participants of the survey, 29 (accounted for 5 % of H8 samples) and 27 persons (accounted for 3 % of H8SP samples,) reported they didn't use the out-patient service when they had illness in the last two weeks before survey. 103 (16%) and 64 persons (7%) reported they

didn't use hospitalization service despite of physician's recommendation in H8 and H8SP towns. Table 3 shows the reasons why they didn't use these services. The leading reason among those factors was financial difficulties (80% for out-patient services and 92% for in-patient services), followed by inconvenient movement and without family member's accompaniment (28% for in- patient services, and 5% for out-patient services). Poor transportation and remote distance (11 % for out-patient services and 10% for in-patient services), also limited their health services utilization to some degree.

**Table 3:** Reasons for non-use of health services when needed (%)

Reasons	Non-user of out-patient services (N = 56)		Non-user of in-patient services (n = 167)	
	
	N	%	N	%
Financial difficulties	45	80	1	92
Inconvenient	3	5	4	28
movement, without				
accompaniment				
Poor transportation and	6	11	1	10
remote distance				
Without effective	2	4	1	7
medical treatment				
Low medical quality	--	--	2	1
Illness not serious	8	14	--	--
Others	3	5	9	5

Note: Reasons for non-use of health services could be chosen by multiple choices

### Influencing factors associated with service utilization

Table 4 shows the influencing factors on service utilization in pilot project areas. In terms of in- patient services, poor families of H8SP towns were less likely to use hospital service (OR = 0.7,95%CI (0.5, 1.0)) compared with H8 towns after adjusting for the other independent variables. Awareness of MFA package (OR = 1.7, 95%CI (1.1, 2.8)) and presence of chronic disease (OR = 3.7, 95%CI (2.4, 5.7)) had significant association with hospitalizations. Factors of marital status and age presented marginal significance on hospitalization use. The analysis results indicated that distance to medical centre had no significant effect on hospital services use (OR = 1.0, 95% CI (0.7, 1.5)).

**Table 4:** Major influencing factors on health service utilization in project areas

Factors	Physician visits※ (n = 190)		Hospitalizations※ (n = 1494)	
	
	OR (CI, 95%)	P	OR (CI, 95%)	P
Type of benefit package (H8/H8SP)	1. 2 (0.8, 1.8)	0.338	0.7 (0.5, 1.0)	0.048
Gender (male/female)	0.8 ( 0.5, 13)	0.476	1.1 (0.7, 1.6)	0.619
Marital status (married/widowed+ divorced)	1.2 ( 0.6, 2.2)	0.561	0.8 (0.5, 1.4)	0.458
Marital status (unmarried/widowed+divorced)	0.9 (0.4, 2.0)	0.857	0.5 (0.2, 1.1)	0.079
Age yr (15-39.9/60+)	1.1 (0.6, 2.1)	0.803	1.5 (0.9, 2.7)	0.138
Age yr (40-59.9/60+)	1.5 ( 0.9, 2.7)	0.151	1.5 (1.0, 2.4)	0.061
Education (Illiterate/junior high school+)	0.6 (0.2, 1.6)	0.321	1.0 (0.5, 1.8)	0.904
Education (Primary school/junior high school+)	0.7 (0.3, 1.8)	0.519	0.9 (0.5, 1.6)	0.720
Disability status (presence/absence)	0.8 (0.5, 1.2)	0.259	1.0 (0.7, 1.6)	0.837
Economic status (Extremely poor/relatively poor)	0.6 (0.4, 0.9)	0.017	1.2 (0.8, 1.8)	0.377
Distance to medical centre (≤3 km/>3 km)	1.5 (0.9, 2.2)	0.087	1.0 (0.7, 1.5)	0.953
Awareness of MFA detailed package (Yes/no)	1.2 (0.7, 2.6)	0.487	1.7 (1.1, 2.8)	0.021
Presence of Illness in last 2 weeks (presence/absence)	--	--	1.4 (0.9, 2.1)	0.119
Presence of chronic disease (Presence/absence)	1.0 (0.6, 1.4)	0.852	3.7 (2.4, 5.7)	0.000

※:Binominal regression with log-link model was fitted using PROC GENMOD

Odds ratio in the table was adjusted for the other remaining independent variables

In terms of influencing factors on outpatient utilization among the respondents who reported their illness in the last 2 weeks, economic status (extremely poor/relatively poor OR = 0.6, 95%CI (0.4, 0.9)) was the important predictor for out-patient service use, which indicated that MFA enrollees with relatively better economic situation were more likely use out-patient services. Distance to the medical centre only showed a marginal significance (OR = 1.5, 95% CI (0.9, 2.2)). As shown in Table 5, the most important factor that influencing frequency of MFA use was

**Table 5:** Major influencing factors on frequency of MFA use in project areas

Parameter	Estimate	S.E.	P
Fixed Effect			
Intercept γ 00	-1.93	0.36	0.0018
Type of package(H8SP/H8) γ01	1.17	0.22	0.0017
Gender (male/female)	0.04	0.05	0.3794
Age, yr	0.01	0.002	0.0001
Education (Illiterate/junior high school+)	-0.05	0.07	0.5397
Education (Primary school/junior high school+)	-0.02	0.06	0.7593
Marital status (Married/widowed+ divorced)	0.09	0.07	0.2471
Marital status (Unmarried/widowed+ divorced)	0.02	0.09	0.8317
Disability status (Presence/absence)	-0.04	0.05	0.4921
Distance from home to the nearest designated medical centre (km)	0.02	0.01	0.8361
Economic status (Extremely poor/relatively poor)	0.01	0.05	0.8151
Awareness of MFA detailed package (yes/no)	0.21	0.05	0.0059
Presence of Illness in last 2 weeks (Presence/absence)	0.23	0.07	0.0119
Presence of chronic disease (presence/absence)	0.31	0.05	0.0006

Variance component			

Var (μ0j ) σ^2^μ 0	0.09	0.05	0.0484
Var (eij) σ^2^	0.68	0.03	0.0001

Model Fit Statistics	-2LL = 3681.2; AIC = 3713.2; AICC = 3713.6; BIC = 3714.5		

Note: Two-level Linear Multilevel model was fitted using SAS PROC MIXED

type of benefit package (H8SP/H8, β = 1.17), which suggested that poor families in H8SP towns had much higher frequencies of MFA use than those in H8 towns. Other factors that also had significant associations with frequency of MFA use were presence of chronic disease (β = 0.31), presence of illness in last 2 weeks (β = 0.23), awareness of MFA package (β = 0.21), age (β = 0.01). All these factors were adjusted for other independent factors, which meant factors of MFA enrollees lived in H8SP town; poorer health status, better awareness of MFA benefit package and much senior age were strong predictors for higher frequency of MFA use.

### Financial burden of MFA enrollees caused by service utilization

Table 6 presents the factors associated with financial burden of MFA cardholders caused by service utilization, which was measured by amount of money borrowed for illness treatment in the last year. We modeled this dependent variable by categorizing medical debt into two groups according to the amount of medical debt (lower than 500 RMB, equal or higher than 500 RMB). No significant difference existed in presence of substantial medical debt between the two benefit package areas (OR = 0.8, 95% CI (0.6, 1.3)), but factors of gender, age, marital status had significant impact on presence of large medical debts. Besides, the factors of presence of chronic disease (OR = 1.5, 95%CI (1.1, 2.2)) and hospitalization (OR = 4.2, 95%CI (2.4, 6.8)) had strong associations with presence of large amount of medical debt, which indicated that MFA cardholders with chronic disease, male gender, aged 40~59.9 and married status were more likely to borrow large amount money to pay for their hospital services.

**Table 6:** Major influencing factors on medical debt of MFA cardholders in project Areas

Factors	Borrowing large amount money(≥500RMB) ※	
	
	OR (CI, 95%)	P
Type (H8SP/H8)	0.8 (0.6, 1.3)	0.281
Gender (male/female)	19.8 (11.5, 34.0)	0.000
Age (15-39.9/60+)	1.4 (0.9, 2.1)	0.387
Age (40-59.9/60+)	2.5 (1.8, 3.7)	0.000
Marital status (unmarried/married)	1.6 (0.9, 2.9)	0.317
Marital status (Widowed+ divorced/married)	0.3 (0.1, 0.5)	0.000
Education (Illiterate/junior high school+)	0.8 (0.5, 1.5)	0.819
Education (Primary school/junior high school+)	1.1 (0.7, 1.8)	0.639
Economic status (Extremely poor/relatively poor)	1.4 (1.0, 2.1)	0.082
Disability status (Presence/absence)	1.0 (0.6, 1.4)	0.874
Distance from home to the nearest designated medical centre (≤3 km/>3 km)	0.8 (0.6, 1.1)	0.321
Awareness of MFA detailed package (Yes/no)	1.5 (1.0, 2.1)	0.045
Presence of Illness in last 2 weeks (Presence/absence)	1.0 (0.4, 2.3)	0.893
Presence of Chronic disease (Presence/absence)	1.5 (1.1, 2.2)	0.036
Physician visit (yes/no)	1.3 (0.6, 3.2)	0.647
High-frequency MFA use (≥2) (yes/no)	1.1 (0.7, 1.7)	0.589
Hospitalization (yes/no)	4.2 (2.4, 6.8)	0.000

※ Binominal regression with log-link model was fitted using SAS PROC GENMOD;

Odds ratio was adjusted for the other remaining independent variables listed in the table.

There was no significant difference in large amount of medical debt rate between the two MFA project areas. Strong association existed between large amount of medical debt and presence of chronic diseases and hospitalization, which suggested that establishment of MFA, had facilitated accessibility of poor families to this new system, and improved service utilization of poor families to some degrees, but its role in reducing poor families' medical debt resulted from disease and hospitalization was still very limited. The original objective of MFA was to provide systematic financial support to poor families, to facilitate the removal of their financial barrier in access to medical services. Our study suggested that there was still great space for further improvement in the design of MFA benefit package to achieve the objectives.

## Discussion

Compared with the original benefit package in H8 towns, the new benefit package of H8SP aimed at further improving the target population's accessibility to health services and overcoming the barriers existed in the pilot project areas through extending coverage of target population, covering out-patient services and reducing the co-payment rate, etc.

In H8SP towns, the coverage of MFA was extended from 5% to 8%~11.3%, which allowed H8SP towns enroll more poor families with relatively better off economic status than that of H8. In fact, the data from ChongQing project office showed that: in Wuxi County, MFA coverage rate in H8 towns was 5.1% while in H8SP towns was 7.5%; in Qianjiang, it was 4.3% in H8 and 8.1% in H8SP respectively. Both of them didn't reach the highest extended target coverage in 2004. However, this extension had partially resulted in significant difference in characteristics of the two samples, and the potential beneficiaries of MFA were enlarged significantly in H8SP towns. In H8SP towns, physician visits among the respondents with illness in last two weeks had increased to some degree compared with H8 towns, whereas, substantial differences existed in frequency of MFA use and hospitalizations after controlling for the need and other confounding factors. The poor families in H8SP towns had made better use of MFA package and less use in- patient services than that of H8 towns.

The indicator of frequency of MFA use was a more sensitive indicator than physician visits in the last two weeks (which often subjected to the influence of acute disease), which could better reflect the overall outpatient services utilization of poor families during last year. Frequency of MFA use had much stronger linkage with the presence of chronic disease in this study.

Chronic diseases are not easily curable, cost much to treatment and bring a lot of economic burden to patients [32]. In poor rural areas, most of the poor families primarily relied on out-

patient treatment and accepted hospitalization only as a last resort because of its high medical cost [15]. Introducing out-patient reimbursement into H8SP benefit package played an important role in encouraging the poor families to use out-patient services when they needed. Adding out- patient reimbursement to the benefit package of H8SP towns significantly increased MFA enrollee's accessibility to the basic health services.

Findings of this study showed that poor people in H8SP had much higher frequencies of MFA use and less use of in-patient services than those in H8. The significant difference between two groups could be partially explained by the reason that poor families' frequent use of out-patient services in H8SP towns could prevent their diseases from getting worse and thus reduced their potential need for hospitalizations. That maybe also contributed to the reduction of percentage of non-use services of MFA cardholders when needed, especially of hospitalization services.

In general, the new package of H8SP had improved the accessibility of MFA enrollee to some degree. Non-use physicians among MFA cardholders with illness in last two weeks of H8SP (26%) was lower than that of H8 (34%), and also lower than the rural average level (43 %) of 2003 national health services survey [26].

However, percentage of non-use but ought-to-use hospitalization in H8SP (54%) and H8 (67%) were much higher than the average rural level (31 %) of 2003 [10]. The reasons for non-use of health services showed that financial difficulty was the leading cause for their giving-up in seeking medical services when needed. Findings of other studies also indicated: financial burden of poor families was still the main barrier to their access of health services [33-36]. Analysis results of the large medical debt of MFA cardholders showed that: in H8SP towns, large amount medical debt was less likely occurred than that in H8 towns, but there is no statistical

significance between them, the debts was strongly associated with hospitalization services and presence of chronic disease, which suggested that, in spite of the extended benefit package of MFA, its financial assistance to poor MFA cardholders was still quite limited.

Although new benefit package of MFA had made great efforts to help poor families to overcome financial obstacle, Reimbursement rate of hospitalization increased from 40~70% to 60 ~ 80%, and for some special cases, services were free. But for most ordinary MFA cardholders, 20~40% co-payments for hospitalization costs still brought a huge economic burden to these poor families.

In addition, regulations of setting ceiling for reimbursement and setting limitations on disease eligibility of MFA had limited poor families to benefit more from MFA. Besides, the poor MFA cardholders with diseases in both H8SP and H8 towns often chose to stand the illness until they became deadly ill, then hospitalization had become their last but also a very expensive resort. This meant the poor families had to borrow large amount of money to afford the co-payment and additional costs for their serious disease due to the ceiling limit of MFA. So unsurprisingly, among those poor families, medical debts had strongly associated with hospitalization use although they had gotten more financial assistance from the benefit package of H8SP than that of H8 towns.

This finding indicated that: there is still space to improve the design of benefit package of MFA Scheme for the policy maker in the future to increase the utilization.

Besides the financial reason, no family member's accompaniment, transportation, and remote distance to health facilities, also played obstructive roles in the accessibility of non-users when they needed. Results of regression analysis showed that: except for type of benefit package (H8SP/H8), among the other variables associated with frequency of MFA use and hospitalization, awareness of MFA was one easily changeable factor. The more poor people learned of this favorable policy, the more likely they used the MFA services. Therefore, promoting access to health care should focus on publicity of these pro-poor policy and program among the poor enrollees.

Our study had some important limitations. The data were collected by survey and therefore subjected to respondent's error in recall. Due to lack of accurate income measuring, we could not directly identify distribution of services utilization among MFA cardholders with different income levels, and couldn't figure out if there exist unequal service uses among MFA cardholders in terms of their ability to pay for co-payment services. Some literature argued that the relatively less poor people were more likely to benefit from services than extremely poor people under the co-payment mechanism of services [8,37,38]. In this study, we introduced an indirect measurement on income level with availability of food for subsistence, and measured financial burden caused by services utilization with medical debt, these variables should need more academic discussion. In addition, due to lack of considering CMS insurance system, we could not able to learn about effectiveness of combination of these two healths security systems on the poor families in project areas.

## Conclusions

In general, this study indicated the new benefit package implemented in pilot H8SP towns had increased the poor population's health service utilization compared with H8 towns to some degree. Out-patient service reimbursement policy encouraged poor families to use service and thus reduced their potential hospitalization need and its financial burden to some extent. But co- payment for in-patient services, ceiling and deductibles for reimbursement, limitations on eligibility for disease reimbursement of MFA, all of them still brought large financial burden to users and was the most important obstacles for poor families' access to health care. Some literature argued that MFA should remove deductibles, reduce co-payments rate and raise ceiling for reimbursement in order to get rid of the huge financial barrier for the poorest families' access to health services [38-40]. Our study results also strongly suggested that: further improvement in design of MFA benefit package is the most important policy option to address these problems in future. Based on our study findings, adding out-patient services to the benefit package of MFA was an important policy option to increase accessibility of the poorest to health care. Improvement of publicity on MFA policy among the poor families was one feasible and operational measure to promote their services utilization.

Recently, Chinese government has issued new health care system reform guideline with extra health budget totaled 850 billions RMB for the reform in following three years. Reform plan highlights universal coverage of NCMS and improvement of MFA in rural China [41]. By now many efforts had made to try to combine these two health security programs together to provide more financial protection for the poor by providing double reimbursements for the eligible beneficiaries [42], which could substantially reduce out-of-pocket payment by the poorest, and thus alleviates their economic burden. The new health reform plan in China will provide more opportunities to explore reasonable and effective benefit package designs to improve accessibility of poor people in rural areas. The related experimental effect of different package design, combined effect of NCMS and MFA for the poorest will be imperative tasks for further research.

## Competing interests

The authors declare that they have no competing interests.

## Authors' contributions

**YHH **participated in design of the study and field survey, performed the statistical analysis, drafted and revised the manuscript. **QHW **was principal investigator of the study, participated in design of the study, field survey, and revised the manuscript. **ZZZ **participated in design of the study, field survey and coordination. **LJG and NN **participated in design of the study and field survey, and **JML **performed the statistical analysis and revised the manuscript. They made equal contribution to the manuscript. **DZ **involved in drafting the manuscript and revising it critically for important intellectual content. All authors read and approved the final manuscript.

## Pre-publication history

The pre-publication history for this paper can be accessed here:

http://www.biomedcentral.com/1472-6963/10/170/prepub
